# 9-year clinical follow-up of patients with ST-segment elevation myocardial infarction with Genous or TAXUS Liberté stents

**DOI:** 10.1371/journal.pone.0201416

**Published:** 2018-08-06

**Authors:** Georgiana-Aura Giurgea, Andrea Heuberger, Jamil Babayev, Susanne Winkler, Oliver Schlager, Irene M. Lang, Mariann Gyöngyösi

**Affiliations:** 1 Department of Angiology, Internal Medicine II, Medical University of Vienna, Vienna, Austria; 2 Department of Cardiology, Internal Medicine II, Medical University of Vienna, Vienna, Austria; University of Tampere, FINLAND

## Abstract

**Objectives:**

This matched-cohort retrospective study investigated the long-term (9-year) safety and efficacy outcomes of patients with ST-segment elevation myocardial infarction (STEMI) and primary percutaneous coronary intervention (pPCI) with Genous (n = 102) versus TAXUS Liberté (n = 101) stents in 2006–2008.

**Background:**

In the era of off-label use of drug-eluting stents for pPCI in patients with STEMI, the use of first-generation Genous stents (endothelial progenitor cell capture stents that have a passive coating and accelerate re-endothelialization) was proposed.

**Methods:**

The primary endpoint was 9-year major adverse cardiac and cerebrovascular events (MACCE), including all-cause death, re-infarction, target vessel revascularization (TVR), and stroke. The secondary endpoints were the separate primary endpoint events at pre-defined time-points (in-hospital, 6 months, and yearly) and stent thrombosis. Time-dependent 9-year composite MACCE, all-cause death, and TVR were compared using Kaplan-Meier estimates and multivariate Cox regression models.

**Results:**

Propensity score analysis confirmed the comparability of the groups. Patients in the Genous and TAXUS groups had 7 and 1 acute definitive stent thrombosis events, respectively (p<0.001). There was a trend towards higher in-hospital MACCE in the Genous group (10.8%) versus the TAXUS group (4.0%). Kaplan-Meier analysis showed that 9-year MACCE was significantly worse in the Genous than in the TAXUS group. The in-hospital, 6-month, 1-year, and 9-year mortality rates were 7.8%, 8.8%, 9.8%, and 23.5% in the Genous group and 2.0%, 3.0%, 4.0%, and 16.8% in the TAXUS group.

**Conclusions:**

Higher peri-procedural, in-hospital, and short-term mortality led to worse outcomes for first-generation Genous stents versus TAXUS Liberté stents for pPCI in STEMI. TAXUS Liberté stents had more favorable 9-year clinical outcomes.

## Introduction

First-generation drug-eluting stents (DESs) reduced the incidence of in-stent restenosis compared to bare metal stents (BMS) in symptomatic coronary arterial disease by inhibiting smooth muscle cell proliferation and preventing intimal hyperplasia. Stents that eluted antiproliferative, immunosuppressive, and anti-inflammatory drugs reduced target vessel failure and consequently improved clinical outcomes [1–3]. However, DESs were also associated with an increased incidence of stent thrombosis, which is mainly attributed to the prolonged endothelialization process and the resulting impairment of vascular healing [4, 5].

Patients with acute ST-segment elevation myocardial infarction (STEMI) were excluded from the first DES trials due to the uncertainty of the thrombotic effect of DESs on vessel patency [3, 6]. Therefore, DESs were used “off-label” in STEMI until late-2000s [7]. In the early and mid-2000s, it was proposed that BMS and Genous stents be implanted during primary percutaneous coronary intervention (pPCI). Notably, the Genous stent was developed in 2001, as the world’s first pro-healing intracoronary stent.

The first-generation Genous bioengineered stent (OrbusNeich Medical Technologies, Fort Lauderdale, FL, USA) was a passive-coated 316L stainless steel stent covered with a biomaterial that contains monoclonal anti-humanCD34+ antibodies, which bind circulating endothelial progenitor cells (EPCs) at the stent surface. When the EPCs were concentrated near the vessel injury site, they helped accelerate endothelialization by differentiating into active endothelial cells. The rapid re-endothelialization of the stented vessel surface limited smooth muscle cell proliferation and theoretically decreased intimal hyperplasia as well as stent thrombosis [8]. Several small and larger cohort and randomized studies that have included patients with stable coronary lesions have demonstrated the efficacy of the Genous stent in reducing target vessel revascularization, restenosis, and stent thrombosis [9–12].

The use of Genous EPC-capturing stents in STEMI was proposed because acute myocardial infarction causes the mobilization of EPCs, which, in turn, can accelerate the healing of the stented vessel injury. Orally administered statins would act to increase the number of circulating EPCs [13]. The idea was that using the Genous stent, with its ability to bind EPCs and its non-eluting passive coating, in combination with the maximum dose of oral statins, might be useful for pPCI in STEMI to prevent target vessel failure. The first cohort and randomized studies that included patients with STEMI who received Genous stents reported favorable short- and mid-term outcomes for clinical endpoints [14, 15].

The aim of this study was to compare the long-term (9-year) clinical outcomes of patients with STEMI who received either Genous or TAXUS Liberté stents during pPCI.

## Methods

### Study design

This study was a retrospective, single-center, 9-year follow-up trial conducted at the Department of Cardiology at the Medical University of Vienna. The study protocol was approved by the Ethics Committee of the Medical University of Vienna and complied with the principles of the Declaration of Helsinki.

### Study population

Between February 2006 and December 2008, consecutive patients with STEMI who received either the Genous or TAXUS Liberté stents during pPCI were included in the analysis. The pPCI procedures were performed in accordance with the contemporary guidelines for the management of patients with STEMI and the choice of stent was left to the physician’s discretion [16]. Use of both BMS and DES was recommended for pPCI without Level A evidence of superiority of DES to BMS, with off-label indication of DES and lack of experience of Genous, as passive coated BMS for pPCI during the study initiation phase.

The inclusion criteria were STEMI according to the STEMI definition guidelines [17], age >18 years, pPCI of the infarct-related artery within 12 hours of symptom onset, and use of either Genous or TAXUS Liberté stenting in the culprit lesion of the infarct-related artery. The exclusion criteria were cardiogenic shock at the time of clinical presentation of the patient, concomitant disease influencing the duration of the dual antiplatelet therapy, or requiring triple antithrombotic treatment (such as chronic atrial fibrillation) and any other disease that limited life expectancy, such as progressive cancer, chronic inflammatory disease, significant valve disease, planned coronary or other cardiovascular surgery, allergy or intolerance for the dual antiplatelet therapy, or significant hematologic disorder.

The following information was recorded: clinical information (age; gender; prevalence of atherosclerotic risk factors, such as diabetes mellitus, hypertension, hypercholesterolemia or smoking; anterior location of the infarction; previous infarction or coronary artery intervention; statin treatment; and door-to-needle time); procedural information (stent size, length, number of stents implanted in the infarct-related artery, pre- and post-dilation, and stent implantation pressure); and follow-up clinical information (stent thrombosis, death, re-AMI, stroke, target lesion and vessel restenosis, target vessel failure, and re-intervention) at the pre-defined time-points.

### Study endpoints

The primary endpoint of the study was the patient-oriented composite clinical endpoint with the incidence of major adverse cardiac and cerebrovascular events (MACCE), including all-cause death, recurrent acute myocardial infarction (re-AMI), target vessel revascularization (TVR), and stroke during the 9-year follow-up, occurring in hierarchical order [18].

The secondary endpoints included the a) separate primary endpoint events at the 9-year follow-up; b) procedural, in-hospital, 6-month, and yearly MACCE rates; c) acute, subacute, and late stent thrombosis (definitive, probable, or possible); d) target lesion restenosis; target lesion revascularization (TLR); target vessel, non-target lesion, and non-target vessel revascularization (non-TVR); and e) major bleeding. All endpoint definitions were in accordance with those of the Academic Research Consortium (ARC) [18].

### Stenting procedure and follow-up

The Genous stent device has been described elsewhere [19]. In cases with multiple lesions, the infarct-related artery was determined based on the coronary morphology, the presence of possible vessel thrombus, and the location of the ECG changes. The infarct-related lesion was treated either with a Genous or a TAXUS Liberté stent. The second lesion stenting or staged procedure was left to the physician’s discretion; all non-target lesions were preferably treated with the same stent type, but this was not mandatory.

Pre- and post-stent dilations were performed at the physician’s discretion. Loading doses of 300 to 600 mg of clopidogrel and 250 mg of aspirin were administered before the procedure or immediately after diagnostic coronary angiography and before the start of PCI. Use of GPIIb/IIIa was left to the physician’s discretion.

During the first year of the study, all patients were treated with dual antiplatelet therapy (DAPT) (a 75-mg dose of clopidogrel plus a 100-mg dose of aspirin daily) in accordance with the guidelines in place at the time. Patients who received a Genous stent were treated with the maximal statin dose immediately after pPCI, irrespective of their previous statin treatment dose or blood lipid levels or existing hyperlipidemia. Follow-up was performed to document all cardiac and non-cardiac events, including a clinical visit, an inspection of the medical record of each patient, or telephone contact after hospital discharge at 6 months, at 1 year, and then yearly for up to 9 years.

### Statistics

Descriptive statistical analyses were performed using continuous variables expressed as mean values ± standard deviations (for parameter with normal distribution) or median with interquartile ranges (for parameter with skewed distribution) and using categorical variables presented as the percent frequencies. For comparisons between groups in the pre-specified sub-analysis, two-tailed t-tests or Kruskal Wallis non-parametric test were used for the continuous variables with normal distribution or skewed distribution, respectively. Chi-squared tests were used for categorical variables.

A propensity score was calculated for each patient after identifying the significant predictors of MACCE by fitting a stepwise logistic-regression model with candidate variables. These variables included patient- and procedure-related predictors, such as age, gender, hypercholesterolemia, hypertension, diabetes, smoking, previous myocardial infarction, number of diseased vessels, and door-to-needle time.

For the primary endpoint, the historical first event was calculated. For the secondary endpoints, all events were calculated, even if a single patient experienced more events. The time-dependent 9-year cumulative incidence rates of composite MACCE, all-cause death, and TVR were compared using Kaplan-Meier estimate supplemented by log-rank statistics. Due to the influence of age at the time of study inclusion (mean age, 62 years) and the 9-year duration of the follow-up, with age and follow-up time expected to have significant influence, the Kaplan-Meier analyses of MACCE and all-cause death were adjusted for age (≥ 62years).

The time dependency of the selected covariates of the Cox regression was tested by creating interactions of the predictors and survival time function, and as expected, the age proved to be a time-dependent covariate. Since this parameter was time-dependent for both groups, we have excluded this parameter from the Cox regression model and performed the proportional hazard assumption to evaluate the effects of potential risk factors on event-free survival. For event-free and survival analyses, the following variables were considered in the multiple regression models: gender, diabetes, hypertension, hypercholesterolemia, smoking, type of implanted stent (Genous or TAXUS Liberté), left anterior descending coronary artery (LAD) as the infarct-related artery, and the number of implanted stents. For TVR, the following parameters were considered as potential predictors: gender, diabetes, hypertension, hypercholesterolemia, smoking, type of stent implanted (Genous or TAXUS Liberté), LAD as the infarct-related artery, proximal lesion, cumulative stent length, and the number of implanted stents. The estimated relative risk (hazard ratio) with 95% confidence intervals were calculated.

The patient-years were calculated as the final follow-up time (days) for the individual patients (considering the time to event) and divided by 365. The number of patients at risk per year was calculated as number of events (MACCE) of the group divided by the patient-year (denominator) and given as percentage.

Post-hoc analysis was performed to analyze the effects of prior statin treatment on the incidences of the following: acute and subacute stent thrombosis (both definitive); in-hospital, 6-month, 1-year, and 9-year death; re-AMI; TVR; and MACCE in patients who received a Genous stent.

We considered p<0.05 to be statistically significant. Statistical calculations were performed with SPSS for Mac version 24.

## Results

### Patients

Between February 2006 and December 2008, 203 patients were included in the study who received either a Genous (n = 102) or a TAXUS Liberté (n = 101) stent. The baseline parameters did not differ between the groups (Table 1).

**Table 1:** Demographic and clinical data of the patients with STEMI receiving either Genous or TAXUS Liberté stents in primary percutaneous coronary intervention (pPCI).

	Genous Group (n = 102)	TAXUS Group (n = 101)	p-value
Age (years)	61.7±11.8	60.8±12.7	0.631
Male n (%)	79 (77.5)	78 (77.2)	0.970
Smoking n (%)	55 (53.9)	58 (57.4)	0.615
Hypertension n (%)	75 (73.5)	73 (72.3)	0.841
Dyslipidemia n (%)	78 (76.5)	78 (77.2)	1
Diabetes mellitus n (%)	18 (17.6)	21 (20.8)	0.570
MI anterior n (%)	48 (47.1)	43 (42.6)	0.521
History of CAD n (%)	38 (38.4)	43 (42.6)	0.546
Previous MI n (%)	14 (13.7)	20 (19.8)	0.246
Previous PCI LAD n (%)	6 (5.9)	5 (5)	0.769
Previous PCI CX n (%)	4 (3.9)	6 (5.9)	0.506
Previous RCA n (%)	4 (3.9)	8 (7.9)	0.227
Previous CABG n (%)	1 (1)	1 (1)	0.994
Prior statin treatment n (%)	76 (74.5)	76 (75.2)	1

Data are given as mean +/- SD or total numbers (percentage) as appropriate.

MI = myocardial infarction, CAD = coronary artery disease, PCI = percutaneous coronary intervention, LAD = left anterior descending coronary artery, CX = circumflex artery, RCA = right coronary artery, CABG = coronary artery bypass grafting

Propensity score analysis revealed a normal distribution of the individual scores in both groups and an expected non-significant difference between the groups (S1 Fig).

### Angiographic and procedural data

The angiographic and procedural data are presented in Table 2. The time from symptom onset to pPCI and the door-to-needle time were similar in the groups. In the Genous and TAXUS groups, 7 and 5 patients, respectively, experienced outdoor cardiopulmonary resuscitation (CPR) before transport to pPCI, with complete hemodynamic stabilization at the time of the clinical presentation.

**Table 2:** Angiographic and procedural data of patients with STEMI receiving either Genous or TAXUS Liberté stents in primary percutaneous coronary intervention (pPCI).

Pre-procedural data	Genous Group (n = 102)	TAXUS Group (n = 101)	p-value
Time from symptom onset to pPCI (Total Ischemic Time) (min)*	54 (39–82)	65 (42–141)	0.172
Door to needle time, (min)*	44 (30–51)	33 (18–89)	0.357
Outpatient CPR	7 (6.9%)	5 (5.0%)	0.368
Pre-procedure TIMI flow 0	70 (68.6%	68 (67.3%)	0.765
Killip Class I or II	92 (90.2%)	94 (93.1%)	0.662
**Procedural data**			
Periprocedural CPR	11 (10.8%)	0 (0%)	<0.001
ReoPro administration	19 (18.6%)	5 (5.0%)	<0.001
Direct stenting n (%)	4 (3.9%)	1 (1.0%)	0.003
Procedural success n (%)	101 (99%)	100 (99.0%)	0.920
Vessel dissection n (%)	1 (1.0%)	2 (2.0%)	0.555
Stent delivery failure n (%)	1 (1.0%)	1 (1.0%)	0.994
Final TIMI flow grade 3	85 (83.3%)	96 (95.0%)	0.260
**Stent data**			
Stent diameter, (mm)	3.1 ± 0.3	3.2 ± 0.5	0.407
Stent length, (mm)	21.1 ± 5.0	21.9 ± 6.0	0.311
Number of implanted stents	1.4 ± 0.5	1.5 ± 0.8	0.112
Diameter of second stent, (mm)	1.1 ± 1.6	1.2 ± 1.5	0.727
Length of second stent (mm)	6.0 ± 8.8	6.6 ± 9.7	0.633
**Angiographic data**			
Target vessel n (%)			0.183
LAD	45 (39.2)	40 (39.6)	
RCA	36 (35.3)	36 (35.6)	
CX/OMCX	21 (20.6)	22 (21.8)	
LAD+CX	0 (0)	1 (1)	
LAD/LM	0 (0)	2 (1)	
Venous bypass graft	0 (0)	0 (0)	
**Number of diseased vessels with significant stenosis**			0.111
One-vessel disease	47 (46.1%)	34 (33.7%)	
Two-vessel disease	34 (33.3%)	39 (38.6%)	
Three-vessel disease	21 (20.6%)	28 (27.7%)	
Complete revascularization	71 (69.6%)	67 (66.3%)	0.654
Staged procedure	5 (4.9%)	8 (7.9%)	0.255

Data are given as mean +/- SD or total numbers (percentage), except *median (interquartile range). pPCI = primary percutaneous coronary intervention, LAD = left anterior descending coronary artery, RCA = right coronary artery, CX = circumflex artery, OMCX = obtuse marginalis of the circumflex coronary artery, LM = left main artery, CPR = cardiopulmonary resuscitation

Table 2 lists the angiographic and procedural data of the patients included into the study. The number of the implanted stents were 1.4±0.4 vs 1.5±0.8 in patients receiving Genous vs Taxus stents. Two patients of each group received stent type other than the original stent type on the infarct-related artery: one Cypher (Cordis, Fremont, California, U.S) and one Endeavor (Medtronic, Minneapolis, MN, U.S.) in group Genous and one rapamycin-coated Yukon (Translumina Therapeutics LLP, New Delhi, India) and 2 Endeavor (Medtronic, Minneapolis, MN, U.S.) stents in the Taxus group, due to missing size or length of the required stent; otherwise, the same stent type was used. Staged procedures were performed with one Endeavor and otherwise Genous stents in the Genous group, and only Taxus stents in the Taxus group.

Stent delivery failure occurred in 1 patient in each group. No significant differences were found regarding the number of implanted stents, stent diameter and length. The lesion pre-dilation balloon diameter (1.6±1.8 vs 1.7±1.0 mm), pre-dilation pressure (6.9±5.5 vs 8.4±5.0 atm), stent inflation pressure (13.9±2.1 vs 14.0±2.5 atm), post-dilation pressure (5.1±7.6 vs 3.9±6.9 atm) were not different between Genous and Taxus groups.

Glycoprotein IIb/IIIa (ReoPro) was administered due to suspected vessel thrombosis of the infarct-related artery in 18.6% and 5.0% of patients who received Genous and TAXUS Libertéstents, respectively (p<0.001). In the Genous stent group, a total of 11 patients (10.8%) experienced acute hemodynamic instability that required peri-procedural CPR due to malignant arrhythmia or acute vessel closure post-stenting (Table 2).

### Clinical outcomes

**Fig 1:**
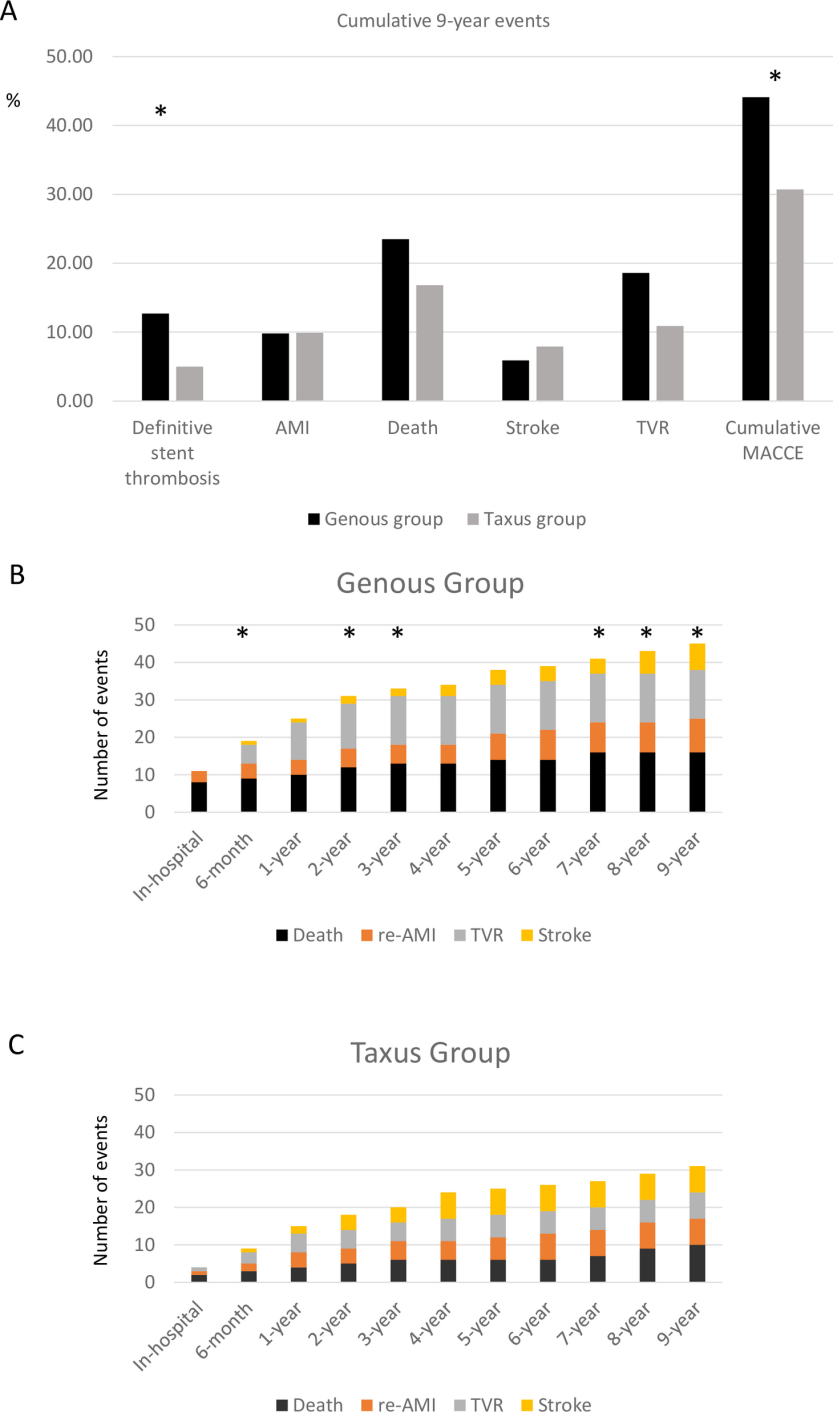
Major adverse cardiac and cerebrovascular events (MACCE) in patients with STEMI who received Genous or TAXUS LIBERTÉ stents in primary percutaneous coronary intervention. **(A)** Primary endpoint: MACCE cumulative 9-year adverse events. **(B)** and **(C)** Secondary endpoints death, repeated acute myocardial infarction (re-AMI), target vessel revascularization (TVR), and stroke in patients who received Genous or TAXUS Liberté stents in primary PCI in STEMI. *p<0.05 between the Genous and TAXUS Liberté groups.

The primary endpoint and the main secondary endpoints (separate primary endpoints events and the procedural, in-hospital, 6-month, and yearly MACCE rate) of the groups are presented in Table 3 and Fig 1.

**Table 3:** List of major adverse cardiac and cerebrovascular events (MACCE, primary endpoint) during the 9-year follow-up in patients with STEMI receiving either Genous or TAXUS Liberté stents during the primary percutaneous coronary intervention (pPCI). **C**umulative MACCE rate is calculated as one event/ patient.

MACCE	Genous group (n = 102)	TAXUS group (n = 101)	p-value
Inhospital MACCE	11 (10.8%)	4 (4.0%)	0.063
Cumulative MACCE at 6 months	19 (18.6%)	9 (8.9%)	0.045
Cumulative MACCE at 1 year	26 (25.5%)	15 (14.9%)	0.059
Cumulative MACCE at 2 years	31 (30.4%)	18 (17.8%)	0.036
Cumulative MACCE at 3 years	33 (32.4%)	20 (19.8%)	0.042
Cumulative MACCE at 4 years	34 (33.3%)	24 (23.8%)	0.131
Cumulative MACCE at 5 years	38 (37.3%)	25 (24.8%)	0.054
Cumulative MACCE at 6 years	39 (38.2%)	26 (25.7%)	0.056
Cumulative MACCE at 7 years	41 (40.2%)	27 (26.7%)	0.042
Cumulative MACCE at 8 years	43 (42.2%)	29 (28.7%)	0.045
Cumulative MACCE at 9 years	45 (44.1%)	31 (30.7%)	0.048

The patient-years were 595 vs 695 for Genous and TAXUS groups, and the patient-at-risk for MACCE rate per year were 7.6% vs 4.5%, respectively.

S1 Table lists the non-serious adverse events, stent thrombosis and bleeding events (secondary endpoints).

In the Genous and TAXUS groups, 7 and 1 patients, respectively, had acute stent thrombosis (p<0.001). There was a trend towards higher in-hospital MACCE in the Genous group versus the TAXUS group (10.8% vs. 4.0%, respectively). At the 6-month follow-up, the MACCE rate was significantly higher in the Genous group (Table 3). The in-hospital, 6-month, and 1-year mortality rates were 7.8%, 8.8%, and 9.8% in the Genous group, and 2.0%, 3.0%, and 4.0% in the TAXUS group. Adverse events were rare after hospital discharge, and the incidence was similar in the two groups. The 9-year mortality was 23.5% vs. 16.8%, and the 9-year cumulative MACCE was 44.1% vs. 30.7% (p<0.05) in the Genous vs. TAXUS stent groups, respectively.

**Fig 2:**
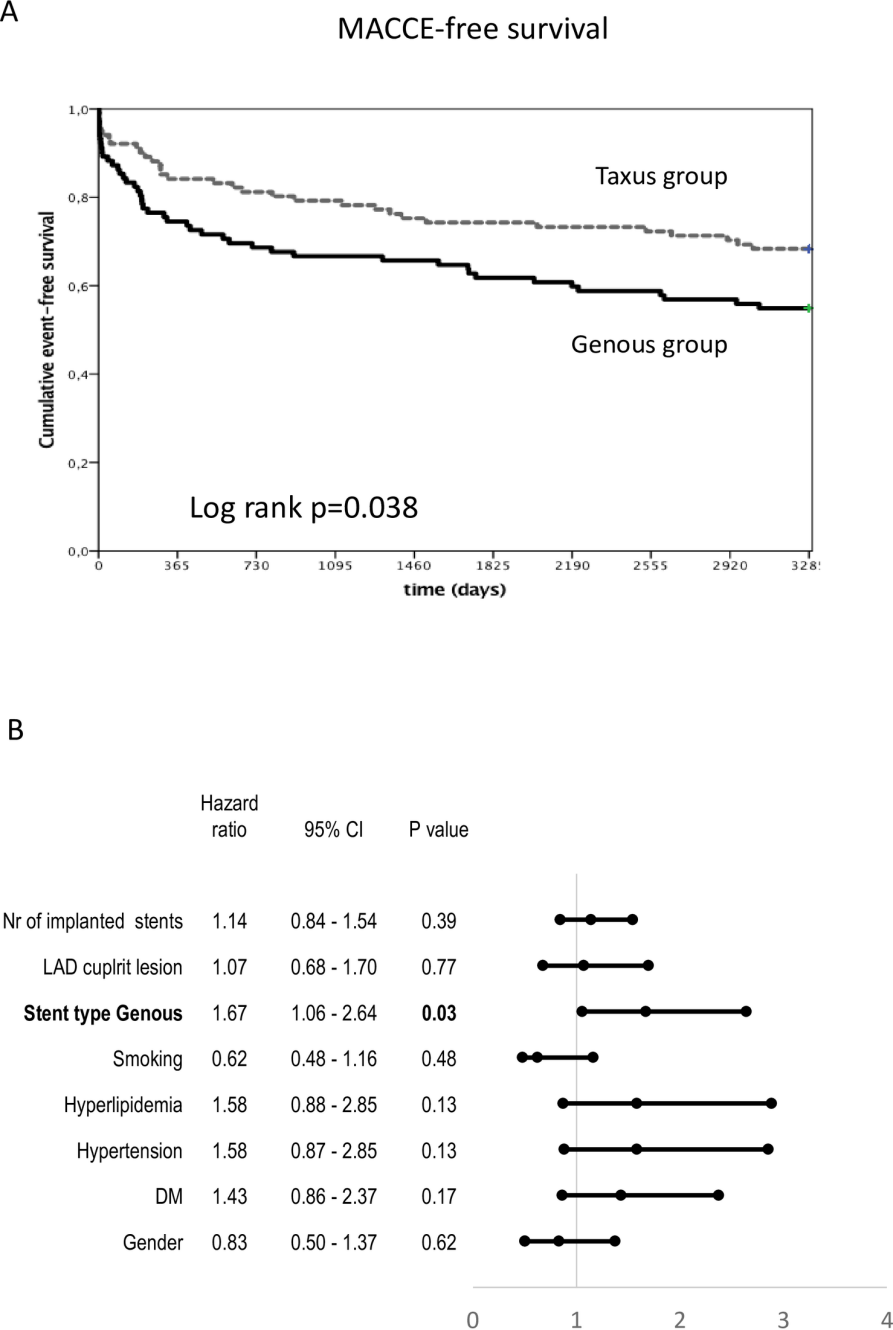
Kaplan-Meier major adverse cardiac and cerebrovascular event- (MACCE)-free survival in patients with STEMI who received Genous or TAXUS Liberté stents in primary percutaneous coronary intervention. **(A)** Cumulative MACCE-free survival. **(B)** Forest plot of MACCE-free survival in the indicated subgroups. CI = confidence interval; DM = diabetes mellitus; LAD = left anterior descending coronary artery; Nr = number.

The Kaplan-Meier 9-year MACCE-free analysis revealed significantly worse outcomes for patients in the Genous group as compared to the TAXUS group (Fig 2).

Cox regression analysis revealed both the use of the Genous stent and greater patient age influenced the time-dependent MACCE (Fig 2).

Adjustment of the Kaplan-Meier MACCE-free analysis with age, however, did not influence the finding that use of the Genous stent was a predictor for MACCE (S2 Fig).

**Fig 3:**
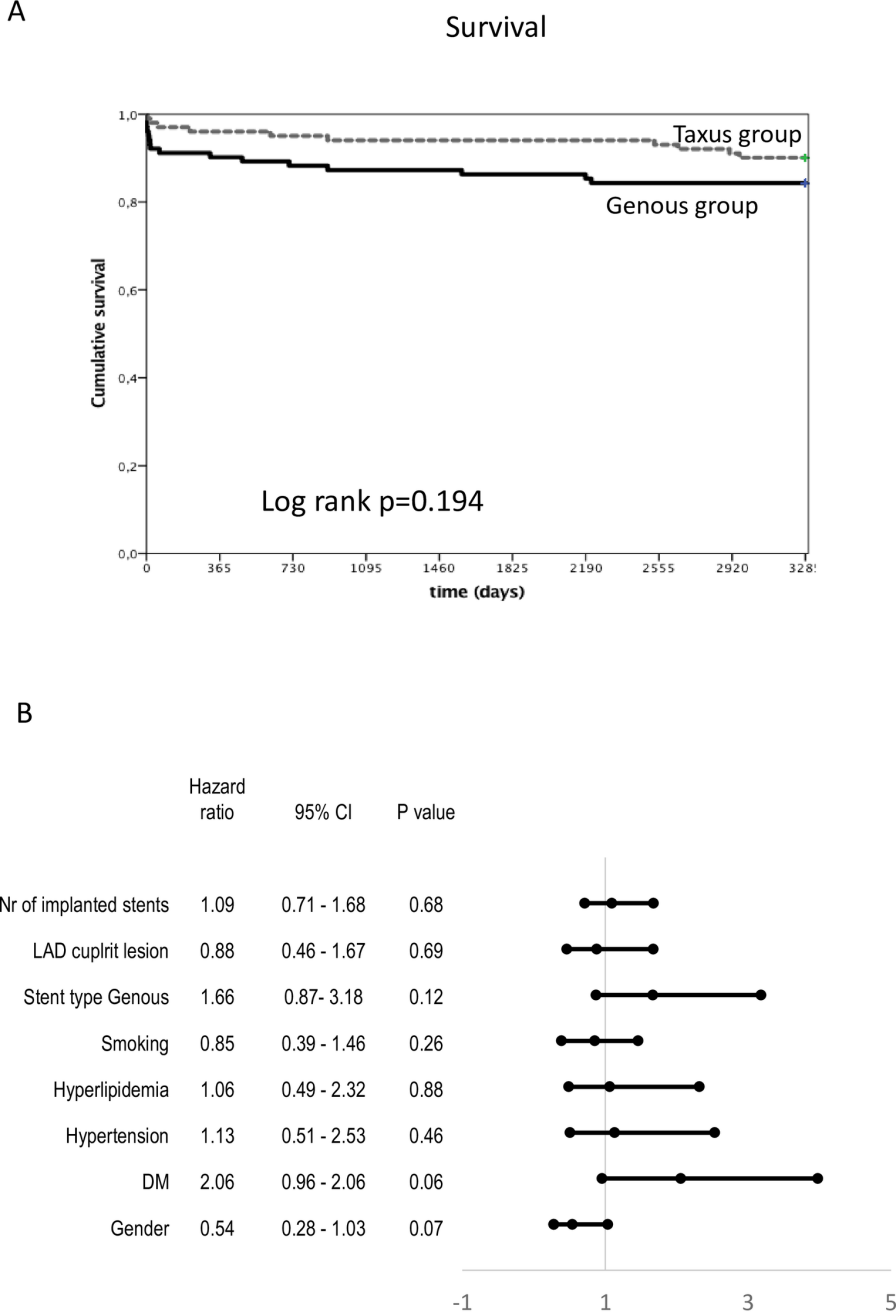
Kaplan-Meier survival analysis in patients with STEMI who received either Genous or TAXUS Liberté stents at the primary percutaneous coronary intervention. **(A)** Cumulative survival. **(B)** Forest plot of survival in the indicated subgroups. CI = confidence interval; DM = diabetes mellitus; LAD = left anterior descending coronary artery; Nr = number.

A trend towards a higher incidence of time-dependent mortality was observed in patients in the Genous group (Fig 3). According to Cox regression analysis, age significantly influenced mortality (Fig 3).

Adjustment with age revealed a trend towards higher mortality in older patients who received a Genous stent as compared with patients with a TAXUS Liberté stent (p = 0.054) (S3 Fig).

**Fig 4:**
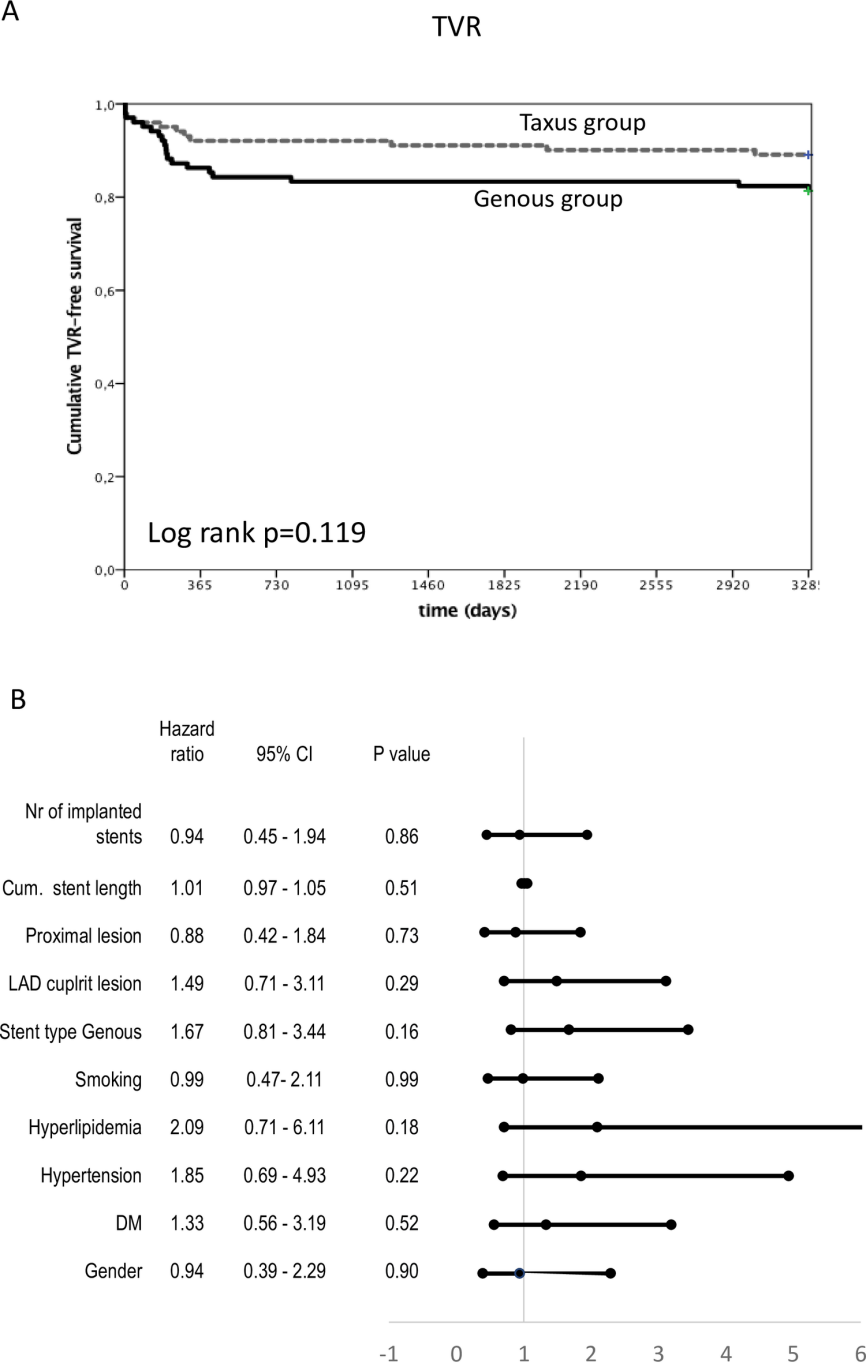
Kaplan-Meier analysis of target vessel revascularization (TVR)-free survival in patients with STEMI who received Genous or TAXUS Liberté stents in primary percutaneous coronary intervention. (A) Cumulative TVR-free survival. (B) Forest plot of TVR-free survival in the indicated subgroups. CI = confidence interval; DM = diabetes mellitus; LAD = left anterior descending coronary artery; Nr = number.

Kaplan-Meier analysis did not reveal differences between the Genous and TAXUS stent groups in terms of time-dependent TVR (Fig 4), which was not influenced either by age, by implantation of the Genous stent, or by other factors (Fig 4).

Notably, target lesion restenosis with consequent target lesion revascularization was significantly higher in the Genous group as compared to the TAXUS group at the 1-year follow-up (S1 Table).

### Subanalyses

Since all patients in the Genous group received maximal dose of statin post-procedure and were treated more often with GPIIbIIIa, which factors might have an influence on the post-procedural clinical events, both of these factors were also entered into the Cox regression model (post-hoc subanalysis). However, none of these factors had effect on the clinical outcome or proved to be an independent predictor for long-term event.

Post-hoc analysis comparing patients in the Genous group with or without prior statin treatment showed similar rates of definitive stent thrombosis, in-hospital deaths, re-AMI, TVR, and MACCE (Table 4).

**Table 4:** List of procedural, in-hospital, 6-month, 1 and 9-year adverse cardiac events in patients with STEMI receiving Genous stents in primary percutaneous coronary intervention (pPCI), with or without prior statin treatment. Each event has been counted; while cumulative MACCE rate is calculated as one event/ patient.

Genous group	Prior statin treatment yes (n = 76)	Prior statin treatment no (n = 26)	p-value
Procedure acute stent thrombosis	2 (2.6%)	0 (0%)	1.00
In-hospital stent thrombosis	3 (3.9%)	0 (0%)	0.568
In-hospital death	7 (9.2%)	1 (3.8%)	0.676
In-hospital re-AMI	3 (3.9%)	0 (0%)	0.568
In-hospital TVR	2 (2.6%)	0 (0%)	1.00
Cumulative 6-month MACCE	17 (22.4%)	3 (11.5%)	0.269
Cumulative 6-month death	7 (9.2%)	2 (7.7%)	1.00
Cumulative 6-month re-AMI	6 (7.9%)	0 (0%)	0.334
Cumulative 6-month TVR	5 (6.6%)	1 (3.8%)	1.00
Cumulative 1-year MACCE	21 (27.6%)	4 (15.4%)	0.293
Cumulative 1-year death	7 (9.2%)	3 (11.5%)	0.69
Cumulative 1-year re-AMI	5 (6.6%)	0 (0%)	0.325
Cumulative 1-year TVR	12 (15.8%)	1 (3.8%)	0.175
Cumulative 9_y MACCE	38 (50%)	8 (30.8%)	0.112
Cumulative 9_y death	19 (25.0%)	5 (19.2%)	0.789
Cumulative 9_y re-AMI	7 (9.2%)	3 (11.5%)	0.712
Cumulative 9_y TVR	16 (21.1%)	2 (7.7%)	0.148
Cumulative stent thrombosis	9 (11.8%)	1 (3.8%)	0.445

AMI acute myocardial infarction

TVR target vessel revascularisation

After pPCI, all patients with a Genous stent took the maximum recommended dose of oral statins. The clinical outcomes of patients who received the TAXUS Liberté stent was not influenced by prior statin treatment (data not shown).

## Discussion

To the best of our knowledge, this is the first study to investigate the long-term (9-year) safety and efficacy of Genous and TAXUS Liberté stents in patients with STEMI who underwent primary PCI. There were four main findings: 1) Patients with STEMI in the Genous group had a higher incidence of acute and subacute definitive stent thrombosis and target lesion revascularization. 2) Patients in the Genous group had a higher incidence of cumulative MACCE at all follow-up times, which was due mainly to a higher death rate, especially in-hospital and at the 6-month and 1-year follow-up. 3) In both stent groups, the event rate was very low after the first year of follow-up in patients who survived the peri-infarct and in-hospital periods. 4) Implantation of the TAXUS Liberté stent in patients with STEMI showed a long-term safety profile with low mortality rates and low adverse cardiac and cerebrovascular event rates.

### Analysis of the Genous stent results in the context of the literature

Tables 5 and 6 summarizes the main data from the literature on Genous stents and lists the main outcomes [8, 11–15, 19–30].

**Table 5:** Main Genous studies.

Author	Study name	Randomiz-ation	Stent types	Patient collective/lesion	Number of patients
Aoki [8]	HEALING-FIM	no	Genous FIM	de novo	16
Co [14]	na	no	Genous	STEMI	120
Kaul [20]	GENAMI	no	Genous	STEMI	11
Lee [15]	na	no	Genous	STEMI	321
Beijk [21]	TRIAS-HR-pilot	yes	Genous vs Taxus	de novo high risk lesion	193
Dammn [19]	e-HEALING	no	Genous Registry	Non-urgent CAD or ACS	4996
Beijk [22]	TRIAS-HR pilot	yes	Genous vs Taxus	de novo high risk olesion	193
den Dekker [13]	HEALING IIB	no	Genous	de novo lesion	100
Low [23]	na	no	Genous	STEMI	489
Klomp [11]	na	no	Genous	unselected, mainly complex lesions	405
Sangiorgi [25]	GENOUS multicenter pilot	no	Genous	de novo secondary vessel 2.5 mm	49
Klomp [12]	TRIAS-HR	yes	Genous vs Taxus	lesions with high risk of restenosis	622
Klomp [24]	E-Healing substudy	no	Genous	elective PCI	3504
Beijk [26]	E-Healing	no	Genous	elective PCI in bifurcation lesions	573
Lehtien [27]	na	no	Genous	LAD lesions	20
Cassee [28]	ARGENTO	no	Genous Registry	elective PCI, allergy to statin	384
Pereia-da-Silva [29]	na	no	Genous	unselected	450
Woudstra [30]	TRIAS-HR	yes	Genous vs Taxus	Lesions with high risk of restenosis	193

FIM: First in Man; HR: high risk; STEMI: ST-segment elevation myocardial infarction; PCI: percutaneous coronary intervention; CAD: coronary artery disease; ACS: acute coronary syndrome; LAD: left anterior descending coronary artery

**Table 6:** Main outcomes of the Genous studies.

Author	Follow-up period	Primary endpoint	Main findings	Conclusions
Aoki [8]	6-month	MACCE	Single patient TVR	feasible and safe
Co [14]	1-year	MACCE	MACCE 1.6%, 4.2%, 5.8% and 9.2% in-hospital, 1, 6 and 12 months	feasible and safe
Kaul [20]	1-year	LLL	high LLL with frequent in-stent restenosis at 8-month	need to be confirmed in larger study
Lee [15]	1-year	MACE	MACE: 30d: 8.1%, 1y: 12.2%, 1Y TVR: 4.4%, 1 acute, 2 subacute STT	Safe, no late thrombosis, good clinical outcome
Beijk [21]	1-year	TVF	higher incidence of TVF and LLL in Genous group	more TVR in Genous group
Dammn [19]	1-year	TVF	TVF signif. more often in elderly patients	Safe
Beijk [22]	2-year	2y safety, efficacy	non-signif. higher rate of TVF (20.4%) as compared to Taxus	non-inferiority to Taxus at 2 years
den Dekker [13]	6-month	6-month in-stent LLL	in-stent restenosis did not decrease	statin increased circulating CD34+ cells 5-6fold without clinical benefit
Low [23]	1-year	MACE	MACE 16% at 34 month, binary restenosis 28% at 1-year	no ST, In-stent restenosis, but not comparable to drug-eluting stents.
Klomp [11]	3-year	TVF	18.3% TVF	3-year safety proven
Sangiorgi [25]	3-month	SCD, AMI, definitive ST	10-days DAPT	10-days DAPT safety proven
Klomp [12]	1-year	TVF	higher TVF compared to Taxus	does not compete with DES
Klomp [24]	12-month	TVF	TVF higher in Western-European than in Asian pts	study reports should include regional outcome differences
Beijk [26]	12-month	TVF	TVF 12.7%, ST 1.7%	favorable clinical outcome, low TVR
Lehtien [27]	30-day	stent coverage by OCT	95% stent coverage	low neointimal hyperplasia, no ST
Cassee [28]	22-month	MACE	DAPT duration (≤15 day or >15 days) did not influence MACE	Safe, and effective regardless of DAPT duration
Pereia-da-Silva [29]	median 36-month (up to 5-year)	MACE	1.8% definitive ST	safe and effective
Woudstra [30]	5-year	TVF	Similar TVF rate at 5-years in Genous and Taxus groups of selected patients	Increase in TVF in Genous is lower between 2–5 years FUP

MACCE: major adverse cardiac and cerebrovascular events; LLL: late lumen loss; MACE: major adverse cardiac events; TVF: target vessel failure; SCD: sudden cardiac death; AMI: acute myocardial infarction; ST: stent thrombosis; OCT: optical Coherence tomography; TVR: target vessel revascularization; DAPT: dual antiplatelet therapy; DES: drug-eluting stent; FUP: Follow-up

Most studies were conducted in patients with noncomplex coronary lesions who underwent elective PCI. The safety and efficacy of Genous stent implantation was first confirmed in the HEALING-FIM study, which included 16 patients with stable or unstable angina or silent ischemia [8] and reported just 1 adverse event, a TVR, at the 9-month follow-up. The subsequent HEALING II study (n = 63) [9, 10] and e-HEALING registry (n = 4939) showed similar beneficial results, with low incidence of TVF and a stent thrombosis rate of 1.1% [31].

However, the first randomized studies in patients with de novo lesions with predicted high-risk restenosis (chronic total occlusion, lesion length >23 mm, vessel diameter <2.8 mm, or any lesion in a diabetic patient) [12, 21, 22] requiring non-urgent PCI failed to reach the primary endpoint and reported higher late lumen loss at 6months and a higher incidence of TVR and TVF at the 1- and 2-year follow-ups. In addition to reporting that the Genous stent proved to be non-inferior in comparison to the TAXUS Liberté stent, the authors found no stent thrombosis in the Genous group.

Co et al. reported the first prospective observational registry data in STEMI patients who received Genous stents and concluded that this procedure is safe, with very low mortality rates of 0.8%, 2.4%, 3.3%, and 3.3% for in-hospital, 1-month, 6-month, and 1-year mortality after STEMI [14]. The only exclusion criterion of this study was cardiogenic shock. Patients received aspirin indefinitely but clopidogrel for only 1 month. Platelet glycoprotein IIb/IIIa inhibitor was used in 14.2% of the patients, and 58% received adjunctive thrombectomy device therapy before stent implantation. In contrast, an Indian pilot study with angiographic follow-up of 11 patients with STEMI reported a high in-stent restenosis rate and high late lumen loss of the infarct-related artery that was treated with the Genous stent [20]. These results were confirmed by the larger angiographic follow-up study by Low et al. that reported a binary restenosis rate of 28% of Genous stent 1-year post-infarction, even though no stent thrombosis occurred [23]. In contrast, implantation of Genous stents in our patients with STEMI resulted in an unusually high rate of periprocedural stent thrombosis, with consequently higher mortality up to the 1-year follow-up. In our study, DAPT was prescribed for 1 year after STEMI; thus, discontinuation of DAPT could not have influenced the 1-year results. Since our STEMI patients who received the TAXUS Liberté stent in the same timeframe showed favorable short- and long-term clinical outcomes, the clearly worse outcomes of the Genous stent patients cannot be explained by differences in study design and population, the use of thrombectomy device, or platelet glycoprotein IIb/IIIa inhibitors.

Notably, our patient population was older than the populations in other studies. Similar to the results of the E-Healing study [19], the 1-year results of the Genous stent in an elderly population showed a significantly higher event rate compared with younger patients. This was mainly driven by higher mortality. After the first year, adverse events were rare in our patients, and it seems likely that such events were due mainly to the multiple comorbidities of the aging patients.

### Comparison of our data with literature data regarding long-term clinical outcomes

It is difficult to compare our data with data from other studies because there are few reports of long-term clinical outcome data of patients with STEMI and primary PCI with either balloon dilation or implantation of any kind of stents. The 9-year mortality of patients with STEMI and primary PCI was 27% in an unselected cohort in the Warsaw STEMI registry, which was an all-comers study that had no exclusion criteria. The registry also included patients who were in cardiogenic shock [32]. TIMI flow 0 or 1 was observed in a high proportion of patients (82%), and 77% of patients received an intracoronary stent in the infarct-related artery. A subgroup of patients in this registry with total ischemic time< 3 h (i.e. the time interval between the onset of symptoms and first balloon inflation) had mortality rates of 7% and 27% at 1 and 9 years, respectively. In contrast, all of our patients received intracoronary stents, and complete or functional occlusion of the infarct-related artery (TIMI flow 0 or 1) was less frequent, with higher post-PCI TIMI 3 flow grade. For comparison, the patients in our study had 1- and 9-year mortality rates of 9.8% and 23.5% in the Genous group and 3.9% and 16.8% in the TAXUS group.

The SIRTAX VERY LATE trial included patients with selected coronary lesions who underwent elective and urgent PCI with first-generation sirolimus- or paclitaxel-eluting stents [33]. That trial reported higher rates of ischemia-driven target lesion revascularization and late and very late definitive stent thrombosis (1.9% and 5.3% at the 1- and 10-year follow-up) with mortality rates of 2.6% and 24.2% at the 1- and 10-year follow-up [33]. Our STEMI patients who received the Genous stent showed a high incidence (12.4%) of definitive stent thrombosis, which included acute, subacute, late, and very late stent thrombosis; of the 12.4%, 9.8% occurred within the first month after primary PCI. This can only be explained in part by STEMI that was accompanied by elevated acute thrombotic risk in the coronary arteries. In contrast, implantation of the TAXUS Liberté, a second-generation DES, resulted in a 5% definitive stent thrombosis rate during the entire 9-year follow-up.

### The Genous endothelial progenitor cell capturing stent: pro-healing technology

In the E-healing registry, it was recommended that patients with planned Genous stent placement receive at least 2 weeks of statin therapy prior to PCI and DAPT for at least 1-month post-procedure with aspirin taken indefinitely. This recommendation was based on the observation that statins increase the peripheral concentration of circulating EPCs [34,35,36]. Patients with low levels of circulating EPCs responded poorly to the Genous stent in the HEALING II study [9,10]. However, the HEALING-IIB study revealed that treatment with the maximum daily dose of atorvastatin 2 weeks prior to and at least 4 weeks after placement of the Genous stent did not reduce the restenosis rate. Subgroup analysis of our cohort who received Genous stents found that prior statin treatment had no influence on the clinical outcome. In fact, complete coverage of the stented artery seems to be independent of the number of the circulating EPCs [13]. The role of EPCs in endothelialization and antithrombotic processes is complex. EPCs can affect the expression or downregulation of thrombotic and coagulation markers, attract CD3 cells, and influence the activity of the vascular smooth muscle cells. However, accelerated endothelialization is still not completely understood, and there are conflicting experimental reports. In addition, intravenous infusion of bone marrow-derived mononuclear cells aggravates the formation of atherosclerotic lesions and contributes to a more vulnerable atherosclerotic plaque that has increased microvascularization and enhanced lipid cores covered by thinner fibrous caps [37]. It seems that formation of a functional endothelial layer from EPCs requires a sequence of signaling events that ranges from cell mobilization, migration and adhesion to cellular differentiation to vascular endothelial cells that form a functional endothelial layer. However, neither our study nor other studies have confirmed that rapid endothelialization has a beneficial effect on stent thrombosis or that it decreases restenosis. It seems likely that factors other than rapid endothelialization play important roles in suppressing thrombus formation.

### Clinical importance of the findings

One strength of the current study was the fairly long observation period of STEMI patients who received either a first-generation Genous stent or a second-generation paclitaxel-eluting stent. Since millions of people have received the Genous and TAXUS Liberté stents and continue to live with them, it is helpful to know that the TAXUS Liberté stent is safe. The occurrence of adverse events after the first year post-STEMI was associated with aging, even in patients who received Genous stents. Due to the reported higher TVF in TRIAS-HR study [12, 21], the Genous stent platform has been changed, from stainless steel to cobalt-chromium, and also to drug-eluting stent system (COMBO Dual Therapy stent, which combines sirolimus with the endothelial progenitor cell-capturing layer), resulting in fewer thrombotic and other cardiac adverse events in clinical studies that are currently on-going or recently published [38,39].

### Limitations

We did not calculate TVF, which is a composite of cardiac deaths, target vessel-related AMI, and TVR. This is because we did not always have the exact cause of death after hospital discharge. However, MACCE is a similar clinical outcome as TVF in terms of cardiac and cerebrovascular events.

This was a retrospective cohort study that was not randomized. However, at the time of patient inclusion, the use of DES was not recommended (although it was not prohibited) for pPCI in STEMI; accordingly, there were no data from randomized clinical studies that used Genous stents in STEMI. Importantly, propensity score analysis confirmed that the Genous and TAXUS Liberté stent groups were comparable. In fact, a strength of our study was that this group of patients collectively represent a real-world clinical scenario, since the study included consecutive patients and only excluded cardiogenic shock or limited life expectancy status in the chosen time frame.

**In conclusion**, first-generation Genous stents used for primary PCI in STEMI had higher procedural and peri-procedural mortality plus higher in-hospital and short-term (1-year) mortality than TAXUS Liberté stents. The TAXUS Liberté stent had more favorable 9-year clinical outcomes.

## Clinical Perspectives

### What`s known?

Several small and larger cohort and randomized studies that have included patients with stable coronary lesions have demonstrated the safety and efficacy of the Genous stent in reducing target vessel revascularization, restenosis, and stent thrombosis.

### What`s new?

This is the first study to investigate the long-term (9-year) safety and efficacy of Genous and TAXUS Liberté stents in patients with STEMI who underwent primary PCI. Patients with STEMI in the Genous group had a higher incidence of acute and subacute definitive stent thrombosis, target lesion revascularization and incidence of cumulative major adverse cardiac and cerebrovascular events. Implantation of the TAXUS Liberté stent in patients with STEMI showed a long-term safety profile with low mortality rates and low adverse cardiac and cerebrovascular event rates.

### What`s next?

The platform of the Genous stent has been changed from stainless steel to cobalt-chromium, and also to drug-eluting stent system, resulting in fewer thrombotic and other cardiac adverse events in clinical studies that are currently on-going.

## Supporting information

S1 Fig Propensity score analysis of patients with STEMI who received Genous or TAXUS LIBERTÉ stents during primary percutaneous coronary intervention. (TIF)

S2 Fig The time to MACCE of patients < 62 years old or ≥62 old years at the time of STEMI. (TIF)

S3 Fig The time to mortality of patients < 62 years old or ≥62 years old at the time of STEMI. (TIF)

S1 Table List of non-serious adverse events (NSAE), stent thrombosis and bleeding (secondary endpoints) during the 9-year follow-up in patients with STEMI receiving either Genous or TAXUS Liberté stents during the primary percutaneous coronary intervention (pPCI). (DOCX)

## Abbreviations

AMI: acute myocardial infarction
BMS: bare metal stents
CPR: cardiopulmonary resuscitation
DAPT: dual antiplatelet therapy
DES: drug-eluting stents
EPC: endothelial progenitor cells
LAD: left anterior descending artery
MACCE: major adverse cardiac and cerebrovascular events
pPCI: primary percutaneous coronary intervention
STEMI: ST-segment elevation myocardial infarction
TLR: target lesion revascularization
TVR: target vessel revascularization
TVF: target vessel failure
