# Supplementary figures and images for: 9-year clinical follow-up of patients with ST-segment elevation myocardial infarction with Genous or TAXUS Liberté stents
Source: PLoS One. 2018 Aug 6;13(8):e0201416. doi: 10.1371/journal.pone.0201416 (PMC6078296; doi:10.1371/journal.pone.0201416)

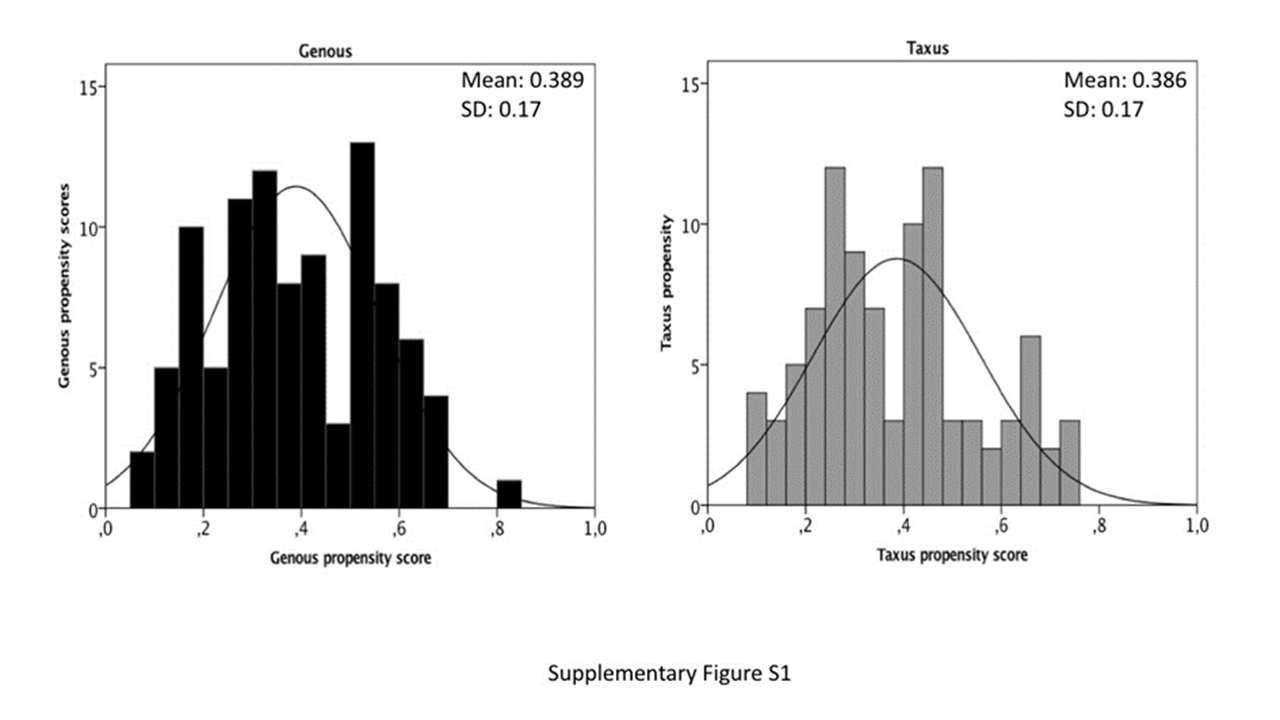

Supplement: S1 Fig — (TIF) [file pone.0201416.s001.tif]

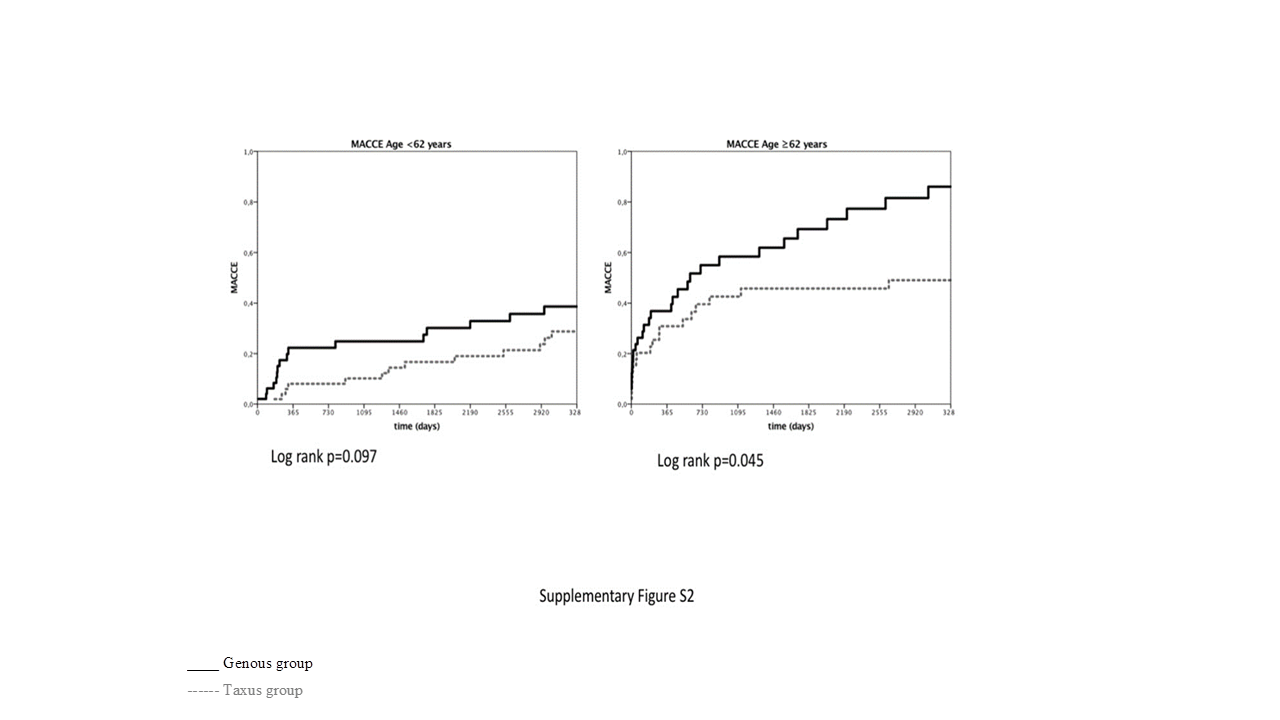

Supplement: S2 Fig — (TIF) [file pone.0201416.s002.tif]

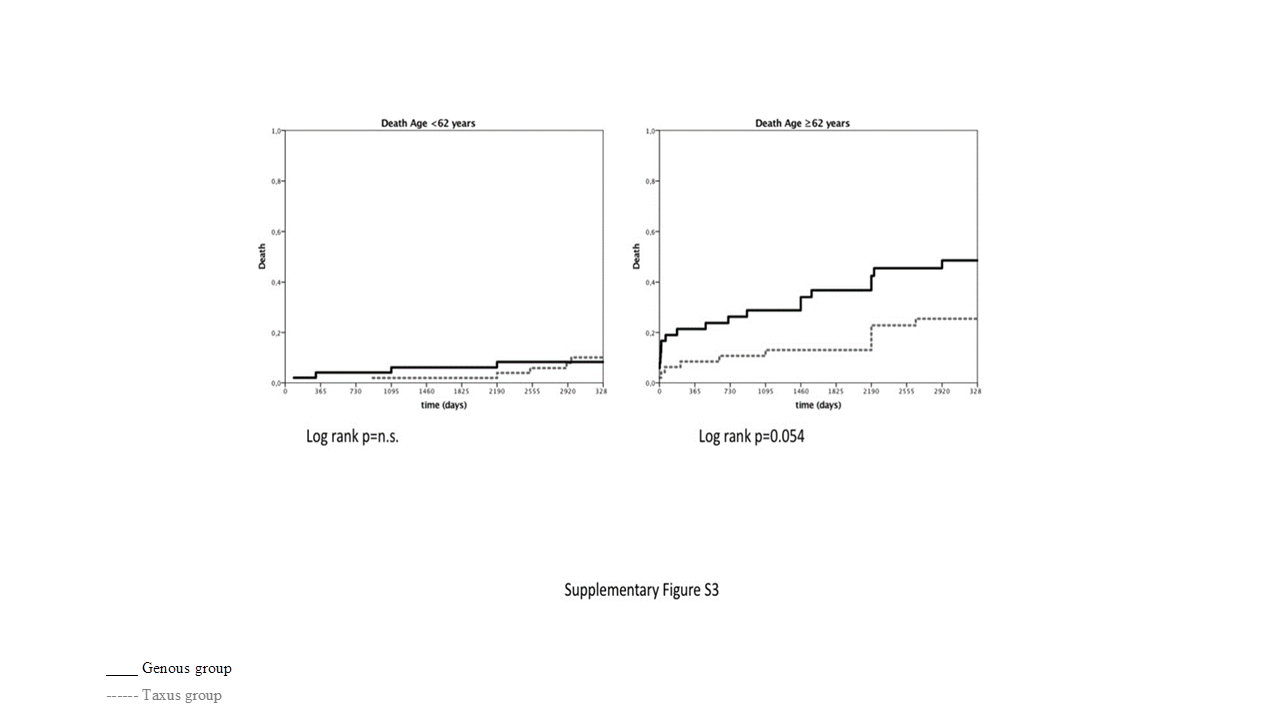

Supplement: S3 Fig — (TIF) [file pone.0201416.s003.tif]
