# Supplementary material for: 9-year clinical follow-up of patients with ST-segment elevation myocardial infarction with Genous or TAXUS Liberté stents
Source: PLoS One. 2018 Aug 6;13(8):e0201416. doi: 10.1371/journal.pone.0201416 (PMC6078296; doi:10.1371/journal.pone.0201416)
Supplement: S1 Table — (DOCX) [file pone.0201416.s004.docx]

**Supporting Information**

**S1 Table. List of non-serious adverse events (NSAE**), stent thrombosis and bleeding (secondary endpoints) **during the 9-year follow-up in patients with STEMI receiving either Genous or TAXUS Liberte stents during the primary percutaneous coronary intervention (pPCI).**

Each event has been counted, while number of NSAE rate is calculated as one event/ patient.

*n* means number of survived patients at the follow-up time

|  | **Genous group (n=102)** | **TAXUS group (n=101)** | **p-value** |
| --- | --- | --- | --- |
| **Inhospital NSAE, *n*** |  |  |  |
| Procedural acute stent thrombosis | 7 | 1 | <0.001 |
| Subacute stent thrombosis | 3 | 3 | 1.00 |
| Bleeding | 2 | 2 | n.a. |
| ***Number of inhospital NSAE*** | ***2/102*** | ***1/101*** |  |
| **FUP 6 months** |  |  |  |
| **Inhospital to 6 months NSAE, *n*** | ***n=94*** | ***n=99*** |  |
| Subacute stentthrombosis | 0 | 0 | n.a. |
| Target lesion restenosis | 4 | 1 | 0.152 |
| Target lesion revascularisation | 4 | 1 | 0.152 |
| Target vessel- non target lesion revascularisation | 1 | 1 | 0.964 |
| Non target vessel revascularisation | 4 | 7 | 0.410 |
| ***Number of NSAEs at 6-months*** | **9/94** | **10/99** |  |
| **FUP 6-12 months** |  |  |  |
| **6-month to 1-year NSAE, *n*** | ***n=93*** | ***n=98*** |  |
| Target lesion restenosis | 10 | 4 | **0.061** |
| Target lesion revascularisation | 6 | 1 | **0.038** |
| Target vessel- non target lesion revascularisation | 3 | 1 | 0.264 |
| Non target vessel revascularisation | 1 | 4 | 0.213 |
| ***Number of NSAEs between 6month and 1-year*** | **12/93** | **14/98** |  |
| **FUP 1 to 2 years** |  |  |  |
| **1 to 2 years events,  *n*** | ***n=92*** | ***n=97*** |  |
| Target lesion restenosis | 3 | 0 | 0.063 |
| Target lesion revascularisation | 2 | 0 | 0.130 |
| Target vessel- non target lesion revascularisation | 0 | 0 | n.a. |
| Non target vessel revascularisation | 1 | 2 | 0.634 |
| ***Number of NSAEs between 1 and 2 years*** | **5/92** | **3/97** |  |
| **FUP 2 to 3 years** |  |  |  |
| **2 to 3 years events, *n*** | ***n=90*** | ***n=96*** |  |
| Target lesion restenosis | 0 | 0 | n.a. |
| Target lesion revascularisation | 0 | 0 | n.a. |
| Target vessel- non target lesion revascularisation | 1 | 0 | 0.278 |
| Non target vessel revascularisation | 1 | 0 | 0.278 |
| ***Number of NSAEs between 2 and 3 years*** | **2/90** | **1/96** |  |
| **FUP 3 to 4 years** |  |  |  |
| **3 to 4 years events,  *n*** | ***n=89*** | ***n=95*** |  |
| Target lesion restenosis | 1 | 2 | 0.657 |
| Target lesion revascularisation | 0 | 1 | 0.354 |
| Target vessel- non target lesion revascularisation | 0 | 0 | n.a. |
| Non target vessel revascularisation | 1 | 0 | 0.277 |
| ***Number of NSAsE between 3 and 4 years*** | **2/89** | **2/95** |  |
| **FUP 4 to 5 years** |  |  |  |
| **4 to 5 years events, *n*** | ***n=89*** | ***n=95*** |  |
| Target lesion restenosis | 0 | 0 | n.a. |
| Target lesion revascularisation | 0 | 0 | n.a. |
| Target vessel- non target lesion revascularisation | 0 | 0 | n.a. |
| Non target vessel revascularisation | 1 | 1 | 0.929 |
| ***Number of NSAEs between 4 and 5 years*** | **2/89** | **2/95** |  |
| **FUP 5 to 6 years** |  |  |  |
| **5 to 6 years events, *n*** | ***n=88*** | ***n=95*** |  |
| Target lesion restenosis | 0 | 0 | n.a. |
| Target lesion revascularisation | 0 | 0 | n.a. |
| Target vessel- non target lesion revascularisation | 0 | 0 | n.a. |
| Non target vessel revascularisation | 0 | 0 | n.a. |
| ***Number of NSAEs between 5 and 6 years*** | **1/88** | **1/95** |  |
| **FUP 6 to 7 years** |  |  |  |
| **6 to 7 years events, *n*** | ***n=86*** | ***n=94*** |  |
| Target lesion restenosis | 1 | 0 | 0.276 |
| Target lesion revascularisation | 0 | 0 | n.a. |
| Target vessel- non target lesion revascularisation | 0 | 0 | n.a. |
| Non target vessel revascularisation | 1 | 0 | 0.273 |
| ***Number of NSAEs between 6 and 7 years*** | **3/86** | **0/94** |  |
| **FUP 7 to 8 years** |  |  |  |
| **7 to 8 years events, *n*** | **n=86** | **n=92** |  |
| Target lesion restenosis | 1 | 0 | 0.269 |
| Target lesion revascularisation | 0 | 0 | n.a. |
| Target vessel- non target lesion revascularisation | 0 | 0 | n.a. |
| Non target vessel revascularisation | 1 | 0 | 0.269 |
| ***Number of NSAEs between 7 and 8 years*** | **1/86** | **0/92** |  |
| **FUP 8 to 9 years** |  |  |  |
| **8 to 9 years events, *n*** | **n=86** | **n=91** |  |
| Target lesion restenosis | 1 | 1 | 0.887 |
| Target lesion revascularisation | 0 | 1 | 0.364 |
| Target vessel- non target lesion revascularisation | 0 | 0 | n.a. |
| Non target vessel revascularisation | 1 | 1 | 0.887 |
| ***Number of NSAEs between 8 and 9 years*** | **2/86** | **3/91** |  |
